# Knockdown of clusterin sensitizes pancreatic cancer cells to gemcitabine chemotherapy by ERK1/2 inactivation

**DOI:** 10.1186/1756-9966-31-73

**Published:** 2012-09-11

**Authors:** Yong Tang, Fenghua Liu, Chunning Zheng, Shaochuan Sun, Yingsheng Jiang

**Affiliations:** 1 Pancreatic Cancer, Key Laboratory of Cancer Prevention and Therapy, Tianjin Medical University Cancer Institute and Hospital, Tianjin, China; 2 Department of general surgery, the affiliated hospital of Jinan central hospital, Shandong university, No105, Jiefang Road, District Lixia, Jinan, 250013, R. P. China

**Keywords:** Pancreatic cancer, Chemoresistance, Gemcitabine, Gene treatment, Clusterin;ERK1/2

## Abstract

**Objectives:**

To study the hypothesis that gemcitabine treatment augments the chemoresistance to gemcitabine by clusterin (sCLU) upregulation. Clusterin inhibition could augment the chemosensitivity of human pancreatic cancer cells by inhibition of clusterin-dependent pERK1/2 activation.

**Methods:**

Clusterin was silenced by serial concentration of OGX-011 transfection in pancreatic cancer MIAPaCa-2 and BxPC-3 cell lines, then treated with serial concentration of gemcitabine. After the cells were treated with OGX-011 for 8 h, the cells were then treated with 5 μM ERK inhibitor PD98059 for 18 h or transfected with a wt-pERK-expressing plasmid into these cells for 24 h, after which the cells were treated with 1.0 uM gemcitabine for 24–72 h. Cell proliferation was determined by MTT. Apoptosis was quantified by flow cytometry,.sCLU and pERK1/2 production was analyzed by western blot, and sCLU mRNA was analyzed by RT-PCR. Xenograft of established tumors was used to evaluate primary tumor growth and apoptosis after treatment with gemcitabine alone or in combination with OGX-011. Phosphorylated ERK1/2 and sCLU levels in tumor tissues were measured by TUNEL analysis.

**Results:**

As detected by MTT and FACS assay, a combination of gemcitabine + OGX-011 reflected the chemotherapeutic sensitivity and increased the gemcitabine -induced apoptosis in MIAPaCa-2 and BxPC-3 cells. Western blotting and RT-PCR analysis revealed that the expression of clusterin was higher in gemcitabine -resistant MIAPaCa-2 cells, however, decreased significantly after pretreatment with OGX-011. Furthermore, the OGX-011 or combination of gemcitabine + OGX-011 decreased the gemcitabine -induced activation of pERK1/2. wt-pERK-re-expression decreased OGX-011+ gemcitabine -induced apoptosis. Finally, OGX-011 in combination with gemcitabine substantially decreased the in vivo tumor growth and promoted apoptosis. Taken together, clusterin confers gmcitabine resistance in pancreatic cancer cells.

**Conclusions:**

Knockdown of clusterin by OGX-011 transfection sensitizes pancreatic cancer cells to gemcitabine by inhibition of gemcitabine -induced clusterin-pERK1/2 activation.

## Introduction

Pancreatic cancer has the worst prognosis of all major cancers, with an overall 5-year survival rate of around 5% [1]. The current clinical standard of care for advanced pancreatic cancer is gemcitabine, a cytotoxic nucleoside analogue. Gemcitabine results in a tumor response rate of 12% and offers a median survival time of 5 months [2]. Unfortunately, this means that the best current treatment offers very modest benefits. Recent studies have indicated that targeted therapies in combination with gemcitabine can have statistically significant benefits [3]. However, the results to date remain meager, and new approaches to improving the effectiveness of gemcitabine are needed. One of the targets considered for combination therapy that has generated wide attention is clusterin [4].

Clusterin, also known as testosterone-repressed prostate message-2 (TRPM-2), sulfated glycoprotein-2 (SGP-2), apolipoprotein J (Apo J) or SP40, is a ubiquitous heterodimeric-secreted glycoprotein of 75–80 kDa. A single-copy gene in humans of nine exons, spanning over 16 kb and located on chromosome 8p21-p12, encodes an mRNA of approximately 2 kb, which directs the synthesis of a 449-amino acid primary polypeptides chain [5]. Recent focus has turned to clusterin as a key contributor to chemoresistance to anticancer agents. Its role has been documented in prostate cancer for paclitaxel/docetaxel resistance [6] as well as in renal [7], breast [8], and lung tumor cells [9]. Moreover, it is abnormally upregulated in numerous advanced stage and metastatic cancers spanning gastric cancer [10], bladder [11], cervical [12], breast [13],ovarian [14], hepatocellular [15], colorectal [16], renal [17], prostate [18], head and neck [19], lung carcinomas [20], melanoma [21]and lymphoma [22].It is noteworthy that only the cytoplasmic/secretory clusterin form (sCLU), and not the nuclear form, is expressed in aggressive late stage tumors, which is in line with its antiapoptotic function [23].

Many reports also document that sCLUc inhibits mitochondrial apoptosis. For example, sCLUc suppresses p53-activating stress signals and stabilizes cytosolic Ku70-Bax protein complex to inhibit Bax activation [24]. sCLUc specifically interacts with conformationally altered Bax to inhibit apoptosis in response to chemotherapeutic drugs [25]. sCLU sliencing alters the ratio of anti-apoptotic Bcl-2 family members, disrupting Ku70/Bax complexes and Bax activation [24,25]. In addition, sCLU increases Akt phosphorylation levels and cell survival rates [26]. sCLU induces epithelial-mesenchymal transformation by increasing Smad2/3 stability and enhancing TGF-β-mediated Smad transcriptional activity [27]. sCLU also promotes prostate cancer cell survival by increasing NF-κB nuclear transactivation, acting as a ubiquitin-binding protein that enhances COMMD1 and I-kB proteasomal degradation via interaction with E3 ligase family members [28]. sCLU sliencing stabilized COMMD1 and I-κB, suppressing NF-κB translocation to the nucleus, and suppressing NF-κB-regulated gene signatures. Thus, sCLU has a key role in preventing apoptosis induced by cytotoxic agents and has the potential to be targeted for cancer therapy.

It has recently reported sCLU was overexpressed in pancreatic cancer tissues and sCLU overexpression confered gmcitabine resistance in pancreatic cancer cells,. Furthermore,sCLU silencing sensitized pancreatic cancer cells to gemcitabine chemotherapy, however the mechanism is still unclear [29].

ERK1/2 is an important subfamily of mitogen-activated protein kinases that control a broad range of cellular activities and physiological processes. ERK1/2 can be activated transiently or persistently by MEK1/2 and upstream MAP3Ks in conjunction with regulation and involvement of scaffolding proteins and phosphatases [30]. There is abundant evidence that survival factors can use the ERK1/2 pathway to increase the expression of several pro-survival BCL-2 proteins, notably BCL-2, BCL-xL and MCL-1, by promoting de novo gene expression in a variety of cell types [31]. Clearly the ERK1/2 pathway can regulate several members of the BCL-2 protein family to achieve cell survival. ERK1/2 signalling can provide protection against chemotherapeutic cytotoxic drugs.

It has shown previously sCLU plays an important role in astrogliosis by stimulating the proliferation of astrocytes through activation of the extracellular signal-regulated kinase 1/2 signaling pathway [32]. Shim and Chou et al. also found significant relation between sCLU and ERK1/2 expression [33,34]. We therefore suggested that sCLU silencing sensitized pancreatic cancer cells to gemcitabine chemotherapy may via ERK1/2 signaling pathway.

sCLU is not a traditional druggable target and can only be targeted at mRNA levels. An antisense inhibitor targeting the translation initiation site of human exon II CLU (OGX-011) was developed at the University of British Columbia and out-licensed to OncoGeneX Pharmaceuticals Inc. OGX-011, or custirsen, is a second-generation antisense oligonucleotide with a long tissue half-life of 7 days, which potently suppresses sCLU levels in vitro and in vivo. OGX-011 improved the efficacy of chemotherapy, radiation, and hormone withdrawal by inhibiting expression of sCLU and enhancing apoptotic rates in preclinical xenograft models of prostate, lung, renal cell, breast, and other cancers [35-39].

In this study, we study the effect of sCLU silencing by OGX-011 on sensitizion of pancreatic cancer cells to gemcitabine chemotherapy, and eluated the mechanisms.

## Materials and methods

### Cell culture

The human pancreatic cancer MIAPaCa-2 cells resistant to gemcitabine and BxPC-3 cells sensitive to gemcitabine [38] were purchased from American Type Culture Collection. They were routinely cultured in DMEM supplemented with 10% fetal bovine serum in a 37°C incubator in a humidified atmosphere of 5% CO_2_.

### Reagents and antibodies

OGX-011 was purchased from OncoGenex Technologies. The antisense oligonucleotides were second-generation 21-mer antisense oligonucleotides with a 2′-O-(2-methoxy)ethyl modification. The antisense oligonucleotide clusterin sequence corresponding to the human clusterin initiation site was 5′-CAGCAGCAGAGTCTTCATCAT-3′ and designated OGX-011 (OncoGenex Technologies). The MEK inhibitor PD98059 was products of Calbiochem (San Diego, CA, USA), Antibodies for sCLU, and phospho-specific or the total form of antibodies against ERK1/2,GAPDH were purchased from Santa Cruz Biotechnology, Santa Cruz, CA, USA).

### Construction of transient transfection with a plasmid expressing human wt-pERK

Total RNA was extracted from PANC-1 cells using TRIzol reagent (Invitrogen, CA, United States), according to the manufacturer’s protocol. The cDNAs were synthesized using the TaKaRa RNA polymerase chain reaction (PCR) Kit (TaKaRa, Japan). A full-length cDNA encoding human wt-pERK was cloned by PCR using 500 ng cDNA as a template and primers containing HindIII and BamHI restriction enzyme sites. The PCR products were ligated into pcDNA3.1 (Invitrogen, CA, United States) to create the plasmid pcDNA3.1- wt-pERK. MIA PaCa-2 and BxPC-3 cells were transfected with the pcDNA3.1 vector or pcDNA3.1- wt-pERK using FuGENE (Roche Diagnostic GmbH, Mannheim, Germany), according to the manufacturer’s protocol.

### Transient transfection

MIA PaCa-2 and BxPC-3 cells were treated with OGX-011(400,800,1000,1200 nM) for 24 h, then the cells were cultured overnight in 6-well plates and transfected with pcDNA3.1- wt-pERK using Lipofectamine Plus (Invitrogen) in 1 ml serum-free medium according to the manufacturer’s instructions. Four hours post-transfection, each well was supplemented with 1 ml of medium containing 20% FBS. Twenty-four hours post-transfection, media were removed and the cells were harvested or treated with gemcitabine for a further 24 hours.

### Western blotting assay

About 25 μg protein was extracted, separated by 10% sodium dodecyl sulfate-polyacrylamide gel electrophoresis (SDS-PAGE), transferred onto polyvinylidene fluoride membranes, and then reacted with primary rabbit antibodies against sCLU(1:100), pERK1/2(1:100) and glyceraldehyde-3-phosphate dehydrogenase (GAPDH)(1:200). After being extensively washed with PBS containing 0.1% Triton X-100, the membranes were incubated with alkaline phosphatase-conjugated goat anti-rabbit antibody for 30 minutes at room temperature. The bands were visualized using 1-step™ NBT/BCIP reagents (Thermo Fisher Scientific, Rockford, IL, USA) and detected by the Alpha Imager (Alpha Innotech, San Leandro, CA, USA).

### RT-PCR assay

The mRNA extraction and RT reaction for synthesizing the first-strand cDNA was carried out according to the manufacturer’s instructions. Primer sequences were below: 5′-CCAACAGAATTCATACGAGAAGG-3′ and 5′-CGTTGTATTTCCTGGTCAACCTC-3′ for sCLU;5′-TGATGGGTGTGAACCACGAG-3′, 3′-TTGAAGTCGCAGGAGACAACC-5′for GAPDH. The PCR conditions consisted of an initial denaturation at 95°C for 3 min, followed by 28 cycles of amplification (95°C for 15 s, 58°C for 15 s, and 72°C for 20 s) and a final extension step of 5 min at 72°C. PCR products were analyzed on a 1.2% agarose gel. The significance of differences was evaluated with Student’s *t*-test. The mean ± SD are shown in the figures. *P* < 0.05 was considered to be statistically significant.

### FACS analysis

To identify the induction of apoptosis, cells underwent propidium iodide (PI) staining and fluorescence-activated cell sorting (FACS) as to the manufacture’s instruction. In brief, cells were plated at a density of 1 × 10^5^ cells/ml. After allowing 24 hours for cell adherence, cells were transfected and/or treated. Cells were collected by gentle trypsinization, washed in phosphate-buffered saline (PBS), pelleted by centrifugation and fixed in 70% ethanol. Immediately prior to staining, cells were washed twice in PBS and resuspended in PBS containing RNAse A (20 μg/ml). Cells were stained with propidium iodide (final concentration 10 μg/ml) for 10 min at room temperature. Samples were analyzed by FACS (FL-3 channel) using a Beckman Coulter Counter Epics XL flow cytometer (Beckman Coulter, Miami, FL, USA). For each sample, 50,000 events were collected and stored for subsequent analysis using EXPO software (version 2.0; Applied Cytometry Systems, Sheffield, UK). The percentage of cells in the sub-G0 phase was quantitated as an estimate of cells undergoing apoptosis.

### MTT assay

Cells were plated at 2 × 10^3^ cells per well in 96-well plates for six days. Cytotoxicity was determined by 3-(4,5-dimethylthiazol-2-yl)-2,5-diphenyltetrazolium bromide assay (MTT, Trevigen, Inc., Gaithersburg, MD) in accordance with the manufacturer’s instructions. Plates were read using a Vmax microplate spectrophotometer (Molecular Devices, Sunnyvale, CA) at a wavelength of 570 nm corrected to 650 nm and normalized to controls. Each independent experiment was done thrice, with 10 determinations for each condition tested. At identical time points,cells were trypsinized to form a single cell suspension. Intact cells, determined by trypan blue exclusion, were counted using a Neubauer hemocytometer (Hausser Scientific, Horsham, PA). Cell counts were used to confirm MTT results.

### Antitumor study

MIAPaCa-2 or BxPC-3 cells (10^7^) were injected into the pancreas of SCID mice. Four weeks after tumor implantation, the mice were assigned to one of the following four treatment groups (n = 10 each): (a) vehicle control; (b) gemcitabine, biweekly treatment 80 mg/kg/injection; (c) OGX-011, biweekly treatment 0.35mg/kg/injection; (d) gemcitabine plus OGX-011, with gemcitabine on Monday and Thursday and OGX-011 on Wednesday and Saturday. All groups received treatment via i.p. injection. Mice in all groups were killed after 5 weeks of treatment. Orthotopic tumors were harvested and weighed.

### In vivo apoptosis assay

Five serial sections (5 um thick) were obtained for each frozen tumor, mounted on glass slides, and then fixed in 4% paraformaldehyde. The first section was processed for H_&_E staining. Apoptosis was evaluated by terminal transferase dUTP nick end labeling [TUNEL] staining using the Apoptag Peroxidase In Situ Detection Kit S7100 [Chemicon] according to the manufacturer’s instructions.

### Statistical analysis

All statistical analyses were performed using the SPSS13.0 software. The results were presented as means ± SD of two-three replicate assays. Differences between different groups were assessed using *X*^2^ or *t*-test. A *P* value of <0.05 was considered to indicate statistical significance.

## Results

### Gemcitabine treatment upregulates sCLU

**Figure 1:**
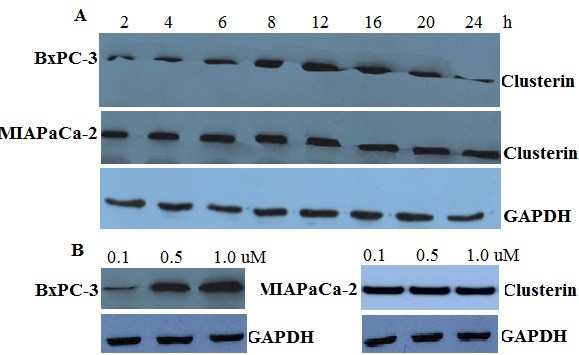
**Induction of sCLU in a time and dose dependent fashion by gemcitabine treatment.A**. Western analysis showing sCLU expression after 2–24 hours treatment with 0.5 nM gemcitabine. Induction of sCLU is evident in chemo-sensitive BxPC-3 cells when treated with high doses of gemcitabine but not in MIAPaCa-2, in which the high levels of sCLU remained unchanged. **B**. Western analysis showing sCLU expression in cell extracts after 12 hours treatment with 0.1-1.0 nM gemcitabine. sCLU increased in gemcitabine -sensitive BxPC-3 cells at different doses. At difference, expression of sCLU was unchanged in the MIAPaCa-2-resistant cells. The data shown are representative of three independent experiments.

To investigate whether upregulation of sCLU expression is a cause or a result of gemcitabine -induced resistance, both MIAPaCa-2(resistant to gemcitabine) and BxPC-3 (sensitive to gemcitabine) cells [40] cells were treated with gemcitabine at 0.5uM for 2–24 h (Figure 1A) or at concentrations 0.1-1.0 uM for 12 h (Figure 1B). Sensitive BxPC-3 cells rapidly responded (sCLU up-regulation peaked by 12 h and began decreasing by 16 h by increasing sCLU expression level under 1.0 uM doses of gemcitabine. MIAPaCa-2 cells already expressing higher sCLU levels, did not further express sCLU following gemcitabine treatment. Considering that changes in sCLU expression seem to be independent of sCLU mRNA, which did not change significantly as indicated by real-time PCR (data not shown). These results suggested that post-translational modification of sCLU may be altered in response to gemcitabine treatment.

### Knockdown of sCLU sensitizes pancreatic cancer cells to gemcitabine chemotherapy

Resistance to anticancer agents is one of the primary impediments to effective cancer therapy. Both intrinsic and acquired mechanisms have been implicated in drug resistance but it remains controversial which mechanisms are responsible that lead to failure of therapy in cancer patients.

**Figure 2:**
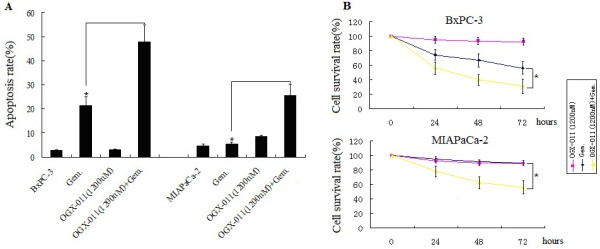
**Targeting sCLU by OGX-011 sensitizes pancreatic cancer cells to gemcitabine treatment.A**, BxPC-3 and MIAPaCa-2 cells were transfected either with OGX-011 (1200nM) and then challenged with gemcitabine dose of 1.0 uM at 24 h. FACS analysis demonstrating that OGX-011 enhanced gemcitabine toxicity in both of the cells. **B**, Comparative viability of MIAPaCa-2 cells and BxPC-3 cells before and after sCLU sliencing. Cells were cultured in 96-well plates, then transfected either with OGX-011. Twenty-four hours after last transfection, cells were treated with gemcitabine. Seventy-two hours after drug addition ,cell viability was estimated. The data shown are representative of three independent experiments (*P < 0.05).

In the present study, MIAPaCa-2 and BxPC-3 cell lines were treated with 1.0 uM of gemcitabine for 24 hours, significant apoptosis (21%) was shown in BxPC-3 cell lines,compared with control(*P* < 0.05). However, in MIAPaCa-2 cells, 1. 0uM of gemcitabine treatment did not induce significant apoptosis (*P* > 0.05). It has shown above only low levels of apoptosis were detected in pancreatic cancer cells following 1.0 uM of gemcitabine treatment. This might be due to the intrinsic and simultaneous induction of clusterin by gemcitabine. Indeed, knockdown of sCLU by 1200 nM OGX-011(maximally reduced sCLU expression) led to a significant increase in gemcitabine-induced apoptosis in both MIAPaCa-2 cells and BxPC-3 cells by FACS analysis (Figure 2A,^*^P < 0.05). However, knockdown of sCLU itself did not affact apoptosis of MIAPaCa-2 cells and BxPC-3 cells (Figure 2A).

On the other hand, cellular viability was studied under experimental conditions similar to this described above. Figure 2B shows significantly less viability of MIAPaCa-2 cells and BxPC-3 cells pre-treated with 1200nM OGX-011(^*^P < 0.05). Together, the aforementioned data indicate that silencing sCLU by OGX-011 enhanced gemcitabine toxicity in the pancreatic cancer cells. Control oligodeoxynucleotide did not have obvious effect on apoptosis or growth in both cells (data not shown).

### ERK inhibitor PD98059 inactivates ERK1/2 in untreated and gemcitabine-treated pancreatic cancer cells

**Figure 3:**
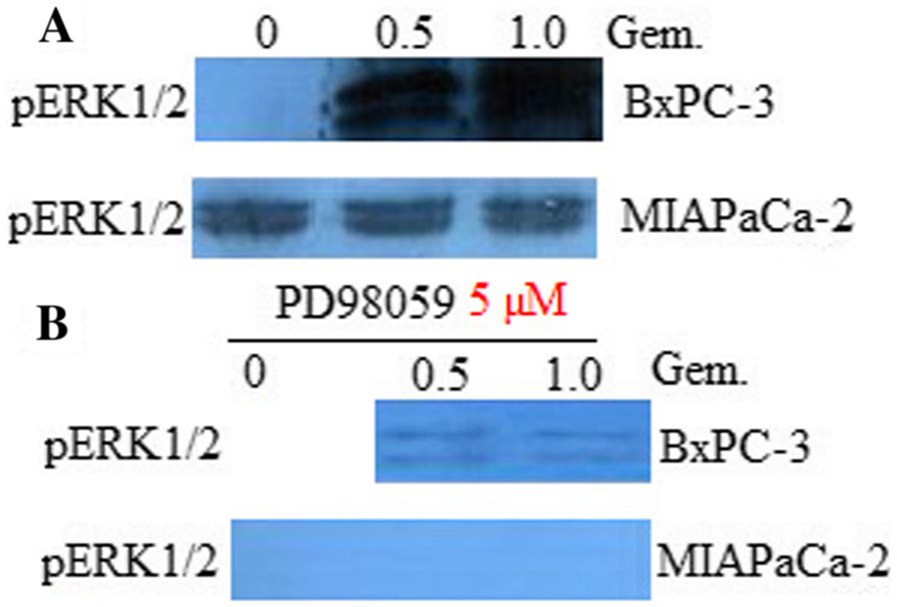
**ERK inhibitor PD98059 inactivate ERK1/2 in untreated and gemcitabine-treated breast cancer cells.A**, BxPC-3 and MIAPaCa-2 cells were exposed to the indicated concentrations of gemcitabine for 18 hr. The cells were then lysed and subjected to WB analysis to monitor pERK1/2 (Thr42/Tyr44) expression as described in Materials and Methods. **B**, BxPC-3 and MIAPaCa-2 cells were exposed (18 hours) to either 5 μM PD98059, 0.5-1.0 μM of gemcitabine, or the combination, after which proteins were prepared and subjected to WB as described above to monitor pERK1/2 expression. For (A) and (B), lanes were loaded with 25 μg of protein; blots were then stripped and re-probed with GAPDH to ensure equivalent loading and transfer. Representative results are shown; two additional studies yielded equivalent results.

Studies were then performed to assess the effects of gemcitabine on ERK1/2 activation in BxPC-3 and MIAPaCa-2 cells. Exposure to 0.5-1.0 μM gemcitabine (18 hr) induced ERK1/2 activation in BxPC-3 cells (Figure 3A).In MIAPaCa-2 cells, 0.5-1.0 μM gemcitabine treatment did not affact ERK1/2 activation (Figure 3A). However, co-administration of the 5 μM ERK inhibitor PD98059 essentially abrogated expression of pERK1/2 in both untreated and gemcitabine -treated BxPC-3(Figure 3B) and MIAPaCa-2 cells (Figure 3B). These findings indicate that in breast cancer cells, 5 μM ERK inhibitor PD98059 essentially abrogate basal ERK1/2 activation as well as gemcitabine -mediated ERK1/2 activation.

### Inactivate ERK1/2 by ERK inhibitor PD98059 sensitizes pancreatic cancer cells to gemcitabine treatment

**Figure 4:**
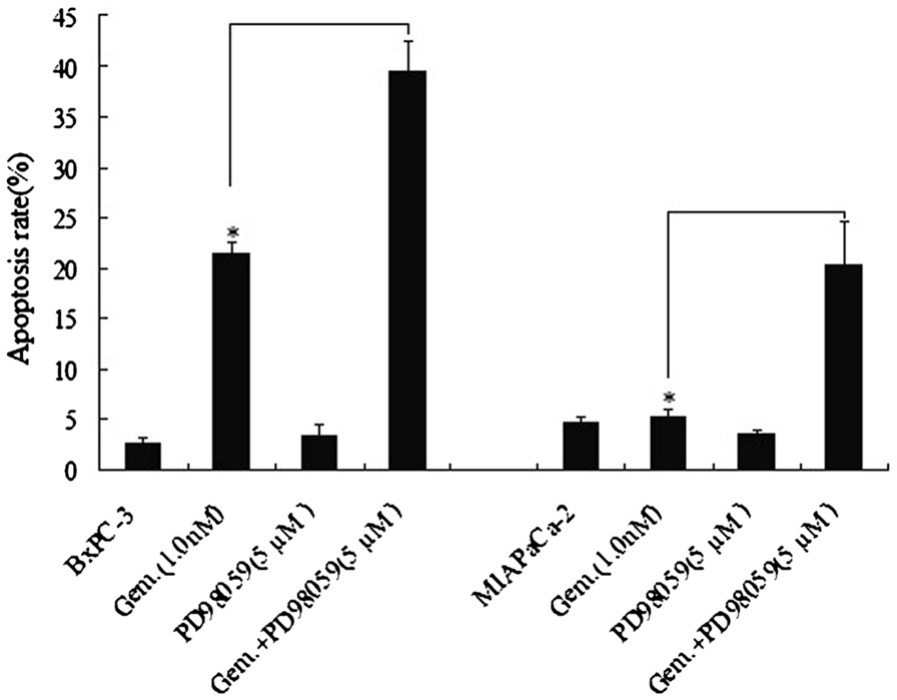
**Inhibition of ERK1/2 sensitizes BxPC-3 and MIAPaCa-2 cells to gemcitabine -induced apoptosis.** BxPC-3 and MIAPaCa-2 cells were treated with 5 μM PD98059 for 18 hours ,then the cells were exposed to 1.0 μM gemcitabine for 24 hours. Gemcitabine -induced cell death was determined by FACS. All values represent the means ± SD for duplicate determinations performed on three separate occasions. * Significantly greater than values obtained for cells cultured in the absence of PD98059; *P* <0.05).

To determine whether ERK1/2 protects pancreatic cancer cells from gemcitabine -induced cell death or not, 5 μM PD98059 was used to inhibit pERK1/2. BxPC-3 and MIAPaCa-2 cells was treated with 1.0 μM of gemcitabine. The results shown both BxPC-3 and MIAPaCa-2 cells were significantly more sensitive to gemcitabine -mediated apoptosis compared to cells exposed to gemcitabine in the absence of PD98059 (*P* < 0.05; Figure 4). It also shows significantly less viability of MIAPaCa-2 cells and BxPC-3 cells pre-treated with 5 μM PD98059 ,then treated with 1.0 nM gemcitabine(data not shown). These findings argue that ERK1/2 inactivation plays a significant functional role in the potentiation of gemcitabine lethality.

### Knockdown of sCLU sensitizes pancreatic cancer cells to gemcitabine treatment via pERK1/2 inactivation

**Figure 5:**
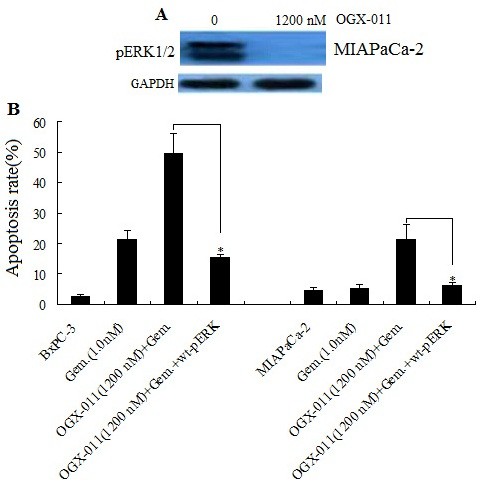
**Knockdown of clusterin sensitizes pancreatic cancer cells to gemcitabine via pERK1/2 inactivation.A**, MIAPaCa-2 cells were treated with 1200 nM OGX-011 for 24 hours, after which proteins were prepared and subjected to Western blot as described above to monitor pERK1/2 expression. **B**, BxPC-3 and MIAPaCa-2 cells were treated with 1200 nM OGX-011 for 8 hours, then a wt-pERK-expressing plasmid was transfected into these cells for 24 hours, after which the cells were treated with 1.0 uM gemcitabine for 24 hours. Gemcitabine -induced cell death was determined by FACS. Representative results are shown; two additional studies yielded equivalent results (^*^P < 0.05).

We first evaluated the effect of sCLU silencing on the pERK1/2 activation in MIAPaCa-2 cells. MIAPaCa-2 cells were treated with 1200 nM OGX-011 for 24 hours. Figure 5A shows significant decrease in pERK1/2 activation in the two cells. BxPC-3 has no basic pERK1/2 expression, so it only used for pERK re-expression. It has shown sCLU silencing itself did not affact apoptosis and growth of MIAPaCa-2 cells and BxPC-3 cells. However, sCLU silencing combined with 1200 nM OGX-011 treatment led to a significant increase in gemcitabine-induced apoptosis in both MIAPaCa-2 cells and BxPC-3 cells by FACS analysi (Figure 2A).We next explored whether pERK re-expression could eliminate the effects of sCLU silencing on gemcitabine-induced apoptosis. BxPC-3 and MIAPaCa-2 cells were treated with 1200 nM OGX-011 for 8 hours, then a wt-pERK-expressing plasmid was transfected into these cells, after transfection for 24 hours ,the cells were treated with 1.0 uM gemcitabine for another 24 hours. While vector transfection did not decrease gemcitabine-induced apoptosis in both MIAPaCa-2 and BxPC-3 cells (data not shown). However wt-pERK-re-expressing in BxPC-3 and MIAPaCa-2 cells significantly decrease in gemcitabine-induced apoptosis (Figure 5B). These data demonstrated knockdown of clusterin sensitizes pancreatic cancer cells to gemcitabine via pERK1/2 dependent pathway.

### In vivo inhibition of tumor growth

**Figure 6:**
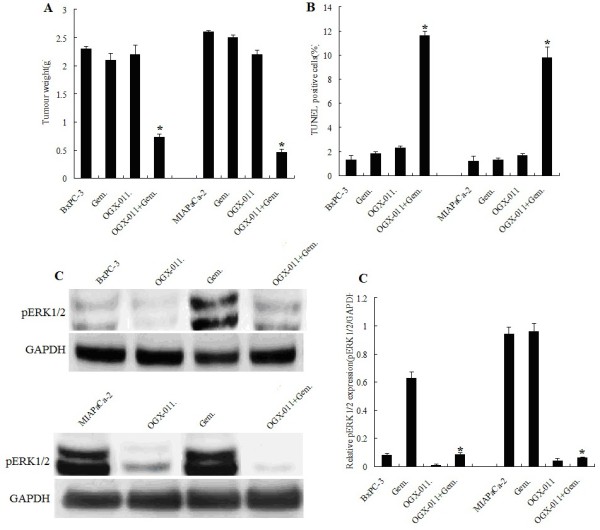
**In vivo inhibition of tumor growth of gemcitabine in combination with OGX-011.A**, Tumor weights in grams (g) in mice treated with the vehicle control, gemcitabine (gem.; 80 mg/kg biweekly, i.p.), OGX-011 (0.25 mg/kg biweekly, i.p.) alone or in combination. Significantly different from the vehicle control group or the gemcitabine-treated group (P <0.01). **B**, TUNEL-positive cells in the vehicle control, gemcitabine or OGX-011 alone or in combination. Significantly different from the vehicle control group (*P < 0.01). **C**, Effects of OGX-011 on tumor tissues in vivo. Representative Western blots showing the levels of pERK1/2 in the vehicle control, gemcitabine or OGX-011 alone or in combination. Similar results were obtained from four separate animals in each group. Significantly different from the combined group or the gemcitabine-treated group (*P <0.01)

Four, two, and three deaths were noted in the vehicle control, gemcitabine-, and OGX-011-treated groups, respectively, before the end of the 5-week treatment period because of large tumors. Conversely, all mice receiving gemcitabine and OGX-011 in combination were alive and exhibited a healthier appearance. Orthotopic tumors were dissected free of surrounding normal tissues and weighed. As shown in Figure 6A, gemcitabine alone did not significantly reduced tumor weights in BxPC-3 and MIAPaCa-2 cells compared to the controls,however, gemcitabine in combination with OGX-011 significantly reduced tumor weights by 5-fold (*P* < 0.001) in MIAPaCa-2 cell relative to the vehicle control, and 3-fold (*P* < 0.001) in BxPC-3 cell relative to the vehicle control. The further decrease in tumor weights observed in the combination treatment group was significantly different from the gemcitabine monotherapy group (*P* < 0.001). OGX-011 alone failed to inhibit tumor growth.

To investigate if the mechanisms involved in the induction of apoptosis in targeted lesions of tumor xenografts represented a phenotypic response of BxPC-3 and MIAPaCa-2 tumors, the TUNEL assay was performed. Representative results are shown in Figure 6B. In the combination treatment groups of BxPC-3 and MIAPaCa-2 tumors, TUNEL-positive cells in tumor sections presented with fragmented nuclei. As shown in Figure 6B, gemcitabine (80 mg/kg) or OGX-011 alone did not produce significant increases in apoptosis compared with the vehicle control. However, the extent of apoptosis was significantly increased by 5-fold (*P* < 0.002) in MIAPaCa-2 tumors ,and 3-fold (*P* < 0.001) in BxPC-3 tumors, treated with gemcitabine and OGX-011 in combination.

To determine whether inhibition of Clusterin by OGX-011 enhances sensitivity to gemcitabine via pERK1/2 inactivation, we detected the pERK1/2 expression by western-blotting assay. As shown in Figure 6C, gemcitabine treatment did not activate pERK1/2 in the MIAPaCa-2 tumors, and gemcitabine treatment signicantly activated pERK1/2 in the BxPC-3 tumors. However, gemcitabine in combination with OGX-011 significantly inhibited pERK1/2 activation.We therefore think that sCLU sliencing sensitizes pancreatic cancer cells to gemcitabine chemotherapy by inhibiton of ERK1/2 activation.

## Discussion

Pancreatic cancer is one of the most difficult human cancers to treat due to the inability to detect disease at an early stage and the lack of effective therapies. Although there has been some progress in the use of improved diagnostic methods and development of novel targeted therapies, the overall survival rate has not improved over the last decade [39]. The most commonly used chemotherapy for pancreatic cancer, gemcitabine, has modest clinical benefit and may not improve overall survival to a clinically meaningful degree [40,41]. The lack of significant clinical response of pancreatic cancer patients to chemotherapy is likely due to the inherent chemoresistance of pancreatic cancer cells as well as impaired drug delivery pathways [42]. Understanding the underlying mechanisms of drug resistance in pancreatic cancer is critical to develop new effective treatments for this deadly disease.

sCLU expression has been implicated in chemoresistance in several other cancer types [43-45], including pancreatic cancer [29]. Because the resistance of tumor cells to various available chemotherapeutic agents has been one of the major factors leading to poor survival in pancreatic cancer patients, we therefore hypothesized that sCLU confers chemoresistance to pancreatic cancer cells.

In this study, we demonstrated that sCLU was correlated with inherent resistance both in vitro and in vivo. We found that high levels of sCLU in pancreatic cancer MIAPaCa-2 cell line was correlated with gemcitabine resistance, low levels of sCLU in BxPC-3 cells was sensitive to gemcitabine .To demonstrate the role of sCLU in gemcitabine resistance, we manipulated the endogenous level of sCLU in a gemcitabine -sensitive BxPC-3 cell line and a gemcitabine -resistant MIAPaCa-2 cell line. We found that gemcitabine -sensitive BxPC-3 cells became more resistant to gemcitabine when endogenous sCLU expression was up-regulated. Conversely, gemcitabine -resistant MIAPaCa-2 cells became more sensitive to gemcitabine and more apoptotic in vitro and in vivo when endogenous sCLU expression was down-regulated by GOX-011 treatment. These results indicated that high levels of endogenous sCLU were involved in the gemcitabine resistance of ovarian cancer cells.

Acquired drug resistance is also thought to be a reason for the limited benefit of most pancreatic cancer therapies.In the present study, we found treatment by gemcitabine increased sCLU expression in BxPC-3 cells, suggesting that sCLU upregulation is likely to be an adaptative response that mediates chemoresistance.We also investigated whether anticlusterin treatment sensitized BxPC-3 cells to gemcitabine.

GOX-011 efficiently inhibited sCLU expression in BxPC-3 cell lines, and this activity was associated with a increase in cell apoptosis in gemcitabine-treated BxPC-3 cells in vivo and vitro. This was indicated that increased sCLU, expression was correlates with gemcitabine resistance in pancreatic adenocarcinoma cells. These results provide preclinical proof of principle for the use of OGX-011 as a novel therapeutic strategy for gemcitabine resistance in the treatment of pancreatic cancer.

Though sCLU confers gmcitabine resistance in pancreatic cancer cells, however, the signaling pathway was unclear. ERK activation has been identified as a potential survival pathway in several tumor types [46], and recent studies show that ERKs may also be activated in response to chemotherapeutic drugs [47-50], and pERK1/2 played critical roles in drug resistance [51]. Our in vitro and in vivo studies here indicated that pERK1/2 play significant roles in gemcitabine resistance to pancreatic cancer cells. Most importantly, we demonstrated that blocking pERK1/2 enhanced the chemotherapeutic potential of gemcitabine in pancreatic cancer cells in vitro. ERK1/2 inhibitors in combination with chemotherapeutic drugs might be a better option to treat patients with pancreatic cancer than drugs alone.

It has shown previously sCLU plays an important role in regulating ERK1/2 signal [32-34].We next study whether sCLU silencing sensitized pancreatic cancer cells to gemcitabine chemotherapy may via ERK1/2 signal. Our results shown sCLU sliencing by OGX-011 sensitizes pancreatic cancer cells to gemcitabine treatment,followed by inhibition of pERK1/2 activation. Conversely, transfection with a constitutively active wt-pERK1/2 construct promotes gemcitabine resistance. These data demonstrated sCLU sliencing sensitizes pancreatic cancer cells to gemcitabine via pERK1/2 dependent signaling pathway.

In conclusion, gemcitabine may influence pancreatic cancer behavior via the upregulation of sCLU, which might play a major role in the effects of gemcitabine, protecting pancreatic cancer cells from the effects of gemcitabine. Inherent chemoresistance of pancreatic cancer cells to gemcitabine may be correlated to sCLU. Blocking sCLU, on the other hand, reverses the drug’s unwanted effects on cancer cell apoptosis and survival. In addition, our studies have firmly established a role for sCLU as a cell survival gene that is increased after gemcitabine chemotherapy to inhibit tumor cell death. The inhibition of sCLU, using OGX-011, enhances the cytotoxic effects of chemotherapy agents via pERK1/2 dependent signaling pathway.

## Competing interests

The authors declare that they have no competing interests.

## Authors’ contributions

TY, LFH, ZCN and JYS performed the majority of experiments; SSC and TY designed the study and wrote the manuscript; TY and JYS edited the manuscript. All authors read and approved the final manuscript.
