# Correction: Knockdown of clusterin sensitizes pancreatic cancer cells to gemcitabine chemotherapy by ERK1/2 inactivation

**DOI:** 10.1186/1756-9966-32-42

**Published:** 2013-07-02

**Authors:** Yong Tang, Fenghua Liu, Chunning Zheng, Shaochuan Sun, Yingsheng Jiang

**Affiliations:** 1 Pancreatic Cancer, Key Laboratory of Cancer Prevention and Therapy, Tianjin Medical University Cancer Institute and Hospital, Tianjin, China; 2 Department of general surgery, the affiliated hospital of Jinan central hospital, Shandong university, No105, Jiefang Road, District Lixia, Jinan 250013, R. P. China

## Correction

After the publication of this work [1], we noticed that we had incorrectly used the term ‘OGX-011’. All instances of OGX-011 in the manuscript should be changed to ‘ASO-CLU’, apart from the last paragraph in the Introduction section which should remain as published.

We also noticed in the first sentence of the second paragraph of the Materials and methods section we mistakenly stated that OGX-011 (ASO-CLU) was purchased from OncoGenex Technologies. As ASO-CLU is currently in the clinical testing phase, it is not available for sale from OncoGenex Technologies. The corrected sentence should read:

ASO-CLU was acquired from OncoGenex Technologies.

We apologise to the readers and OncoGenex Technologies for this oversight and any negative effects that may have resulted from it.
